# Automatic segmentation framework of X-Ray tomography data for multi-phase rock using Swin Transformer approach

**DOI:** 10.1038/s41597-023-02734-7

**Published:** 2023-11-20

**Authors:** Hao Chen, Xiaoqi Cao, Xiyan Zhang, Zhenyu Wang, Bingjing Qiu, Kehong Zheng

**Affiliations:** 1 College of Mechanical Engineering, Zhejiang Sci-tech University Hangzhou, Xiasha, 310018 Zhejiang China; 2 Center Sinohydro Bureau 12, Co., LTD., Hangzhou, China; 3 College of Civil Engineering and Architecture, Zhejiang University, Hangzhou, 310058 China; 4 Center for Hypergravity Experimental and Interdisciplinary Research, Zhejiang University, Hangzhou, 310058 China

**Keywords:** Composites, Imaging techniques

## Abstract

A thorough understanding of the impact of the 3D meso-structure on damage and failure patterns is essential for revealing the failure conditions of composite rock materials such as coal, concrete, marble, and others. This paper presents a 3D XCT dataset of coal rock with 1372 slices (each slice contains 1720 × 1771 pixels in *x* × *y* direction). The 3D XCT datasets were obtained by MicroXMT-400 using the 225/320kv Nikon Metris custom bay. The raw datasets were processed by an automatic semantic segmentation method based on the Swin Transformer (Swin-T) architecture, which aims to overcome the issue of large errors and low efficiency for traditional methods. The hybrid loss function proposed can also effectively mitigate the influence of large volume features in the training process by incorporating modulation terms into the cross entropy loss, thereby enhancing the accuracy of segmentation for small volume features. This dataset will be available to the related researchers for further finite element analysis or microstructural statistical analysis, involving complex physical and mechanical behaviors at different scales.

## Background & Summary

Identifying different features and phases within composite rock materials such as coal^1^, concrete^2,3^, marble^4,5^, etc., enables the rational numerical prediction of mechanical properties, failure conditions, and prevention mechanisms of composite rock under complicated loading conditions, yielding accurate prediction results. Current research on the failure prediction mechanism of composite rock materials is mainly based on experimental analyses from the macroscopic perspective, including static compression loading^6^, dynamic loading, and impact loading^7–9^, etc. The limitations of such experiments are that the effective information gained is confined to examining the stress-strain characteristics^10^, particle size distribution characteristics^11^, and surface fracture evolution^12^. Thus, accurate 3D representations of the rock-like material meso-structures, in which the different material phases are segmented and labelled, aid in explaining their failure behaviors through computational simulations.

XCT images provide valuable information about the state of the material in 3D,such as mineral inclusion^13,14^ and their spatial distribution^15,16^, fracture morphology^12,17^, fracture density^18^, and their correlation with macro-scale fracture mechanisms. The development of CT technology and equipment in the field of coal-rock damage is summarized^19,20^, and research progress on CT characterization of coal-rock damage is also overviewed^21,22^. XCT image segmentation refers to the classification of the related pixels into different mineral phases (including pore space, cracks or more detailed subphases), which is one of the most crucial steps to extract useful information for subsequent mechanical property analyses. Due to non-ideal scanning conditions, reconstruction algorithms, and limited CT resolutions, the related scanning experiments might lead to the partial volume blurring (PVB) effect of composite rock materials, which makes extracting internal phases automatically a near to impossible task^23,24^.

Several traditional segmentation methods, such as the edge detection algorithm^25,26^, watershed algorithm^27–29^, graph cut algorithm^30^, and clustering algorithm^31^, have been widely used for segmenting the meso-structures of composite rocks. However, because of weak boundaries among the sub-phases and the blurring effect caused by PVB, the segmentation errors (including over-segmentation, under-segmentation, or even complete loss of small targets) caused by fixed thresholding were inevitable. Several segmentation algorithms have been developed to solve the above problems. For instance, a segmentation method of fractures based on contour evolution and gradient direction consistency was proposed to accurately segment the fracture networks in the sequence of coal rock CT images^32^. The gray level co-occurrence matrix (GLCM) theory was also applied to quantitatively analyze the meso-damage evolution and the fracturing characteristics using the acquired CT images at each scanning stage^33,34^. The tensile fracture behaviors of concrete are captured by Monte Carlo simulations (MCSs) of realistic meso-scale models based on high-resolution micro-scale XCT images^35–37^. From the above analysis, image intensity or image gray cannot provide sufficient information for the accurate segmentation of the related XCT image with low contrast and high noise, particularly different mineral phases with small targets and weak boundaries.

In this regard, the related challenges can be addressed by using a deep learning (DL) approach, particularly convolutional neural networks, which have produced outstanding success in classifying and segmenting images^38,39^. Compared with traditional segmentation methods, the DL models only need to be trained once and then can be used for new datasets with the same size and resolution or similar morphology^40^, thereby significantly reducing computational costs and avoiding training errors. Researchers in the field of medical image segmentation employ DL approaches to solve problems such as tumor segmentation^41^, cell segmentation^42^, lung segmentation^43^, and organ segmentation^44^. In recent years, supervised DL techniques, such as attention mechanism^45^, feature pyramid^46^, and encoder-decoder models^47^, have been widely applied to the semantic segmentation of composite rock materials. The U-Net model^48^ and its variants^49^ were invented based on fully convolutional networks^50^, which have been widely used in the study of porous media, including mineral classification^51,52^, porosity estimation^53,54^, and fluid flow prediction^55^. However, a significant drawback with U-Net and its variants is that continuous pooling operations degrade feature resolution, leading to the loss of spatial information. This may lead to achieved segmentation results that are usually low in accuracy on the boundary and small target identification.

To solve the above-mentioned challenges, we present a new procedure for using Swin Transformer (Swin-T)^56^ to accurately segment XCT images of composite rock materials for accurate characterization to generate digital virtual FE models. Swin-T is a new deep learning network structure based on global feature extraction, which integrates the features of DCNN to extract features from multiple scales. Firstly, the performance of Swin-T was compared with the U-Net and Deeplabv3 + models^57^ using various evaluation indicators. This was followed by the investigation of the effect of various patch sizes, various loss functions, and various networks on the accuracy, loss, and IoU index under the same batch size and the same number of epochs. Finally, the accurate meso-structure reconstruction of composite rock, with a volume fraction of 0.47% cracks, 91.81% coal, 7.19% gangue, and 0.53% pyrite, was carried out, and then the computational model was generated for later mechanical simulation and presented as an example.

## Methods

### Experimental detail

The raw coal samples were collected from Jin-ling Coal Mine located in Henan Province, China, and then cut into the cube sample with a height of 40 mm. The CT-scanned raw images were obtained from the X-Ray Imaging facility (MicroXMT-400) located at Zhejiang University, using the 225/320kv Nikon Metris custom bay. By rotating the sample, XCT projections from different angles of the specimen are collected for computational reconstruction, resulting in 1372 CT slices (each slice consisting of 1720 × 1771-pixel array).

**Fig. 1:**
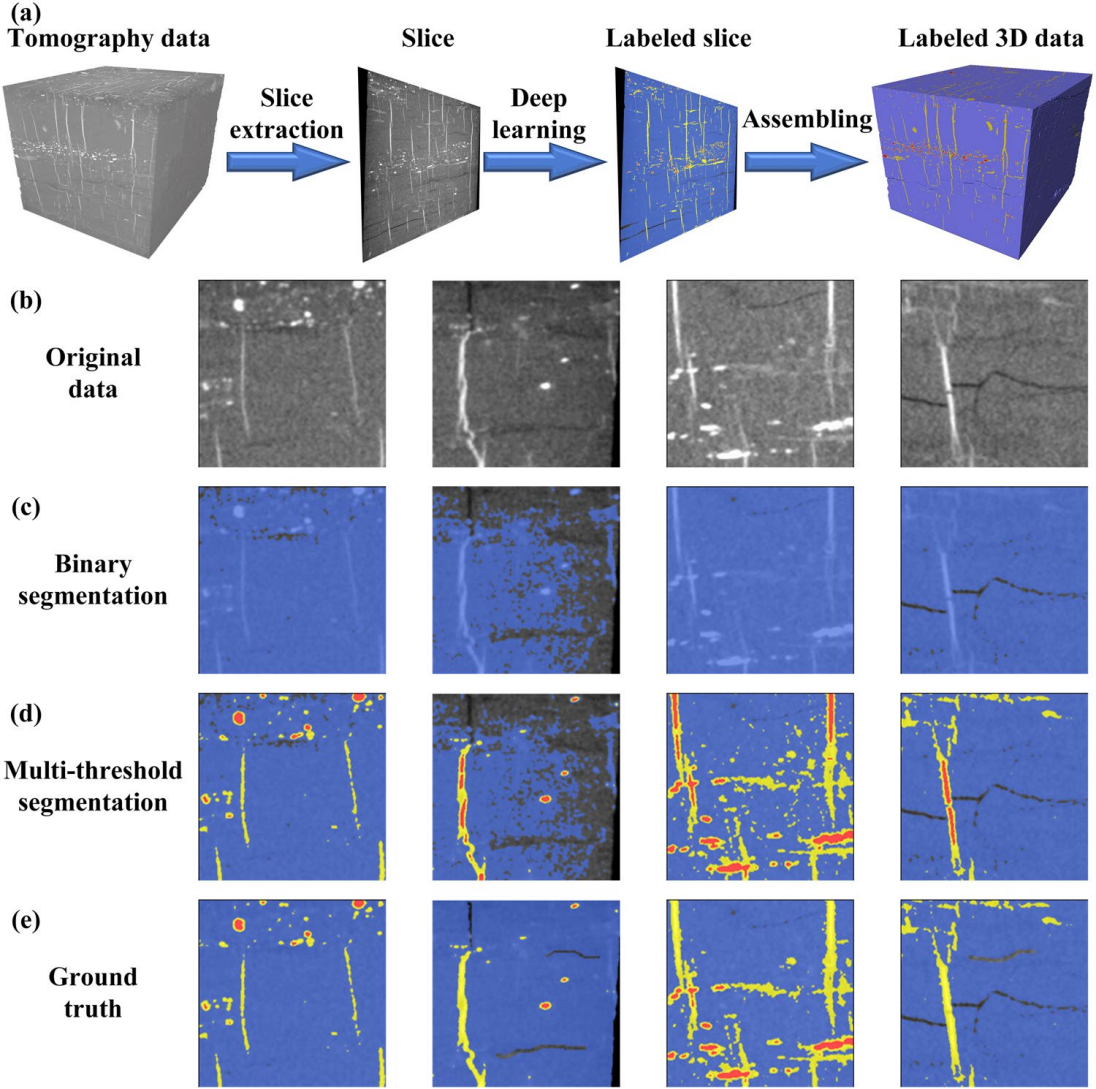
Deep learning-based segmentation and labeling. (**a**) presents the workflow of the deep learning-based segmentation; (**b**–**e**) presents the comparison of traditional segmentation results and the deep-learning segmentation results for a few representative samples. Different colors denote different phases.

In our previous study^58^, the selected coal sample were simplified into coal and gangue phases, without considering the pyrite and void phases. This might further cause inaccurate 3D reconstruction of numerical models. Figure 1 shows the raw images, the segmented binary and four-class images. Particularly, a binary segmentation image shown in Fig. 1b only contains void (black) and solid (white) components. Actually, the void component represents cracks, whereas the solid component can be sub-classified into coal, gangue, and pyrite components, leading to a four-class segmentation image. In the four-class segmentation, cracks, coal, gangue, and pyrite are shown in light gray, blue, yellow, and red, respectively, due to their increasing densities.

As shown in Fig. 1c, traditional segmentation methods often fail to achieve high accuracy and strong adaptability when dealing with all types of CT images with low signal-to-noise ratios. To address this issue, a common solution for deep learning is to provide a pixel-level semantic classification map, where each pixel is labelled with different subphases. The primary challenge in image segmentation of composite rock materials is the lack of ground truth, which complicates the evaluation of the accuracy of a particular image segmentation method because the ground truth is unknown.

The ground truth masks shown in Fig. 1d were manually annotated using the commercial software Avizo 9.0. A total of 158 manually segmented slices were selected as the ground truth datasets by selecting one image every 10 of 1579 images. In the selected 158 ground truth datasets, 143 slices were used as the training datasets, and 15 slices were employed as the validation datasets.

### Data preprocessing

To eliminate noise within the image while preserving the edges and enhancing the contrast, the median and non-local mean filters were applied to the raw grayscale image, and then the bit depth of the raw dataset was reduced from 16 to 8 bit, which aims to reduce the data processing time.

### Data augmentation

Deep learning requires a large amount of training data, which is not always available due to the massive difficulty and labor cost of ground truth annotation for XCT images. A standard technique to increase the size and variances of the training data is to apply a reasonable transformation to the input data randomly.

**Fig. 2:**
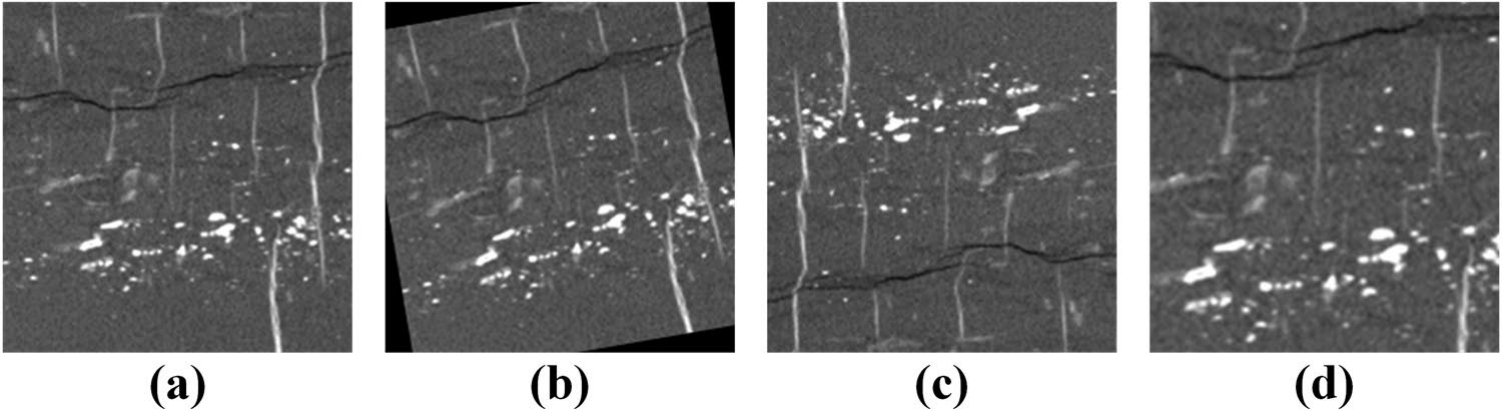
Data augment effect. (**a**) original image (**b**) random rotation (**c**) random flipping (**d**) random scaling.

For the selected typical rock XCT images, the different sub-phases are spatially continuous regions. This implies that the network can easily learn local variations, such as the distribution characteristics of different mineral phases. As shown in Fig. 2, random transformations, such as cropping, flipping^48^, padding^49^, rotation^50^, and other methods were used to expand the volume of the training data, which will mitigate over-fitting problems. We randomly selected some images for data augmentation using the following transformations: scaling in the range [0.8, 1.2], rotation by [−10, 10] degrees, and mirroring along the vertical and horizontal axes. Considering the poor X-ray CT scanning quality, 6% Gaussian random noise was also added to some images to improve the robustness of the method.

### Transformer model

**Fig. 3:**
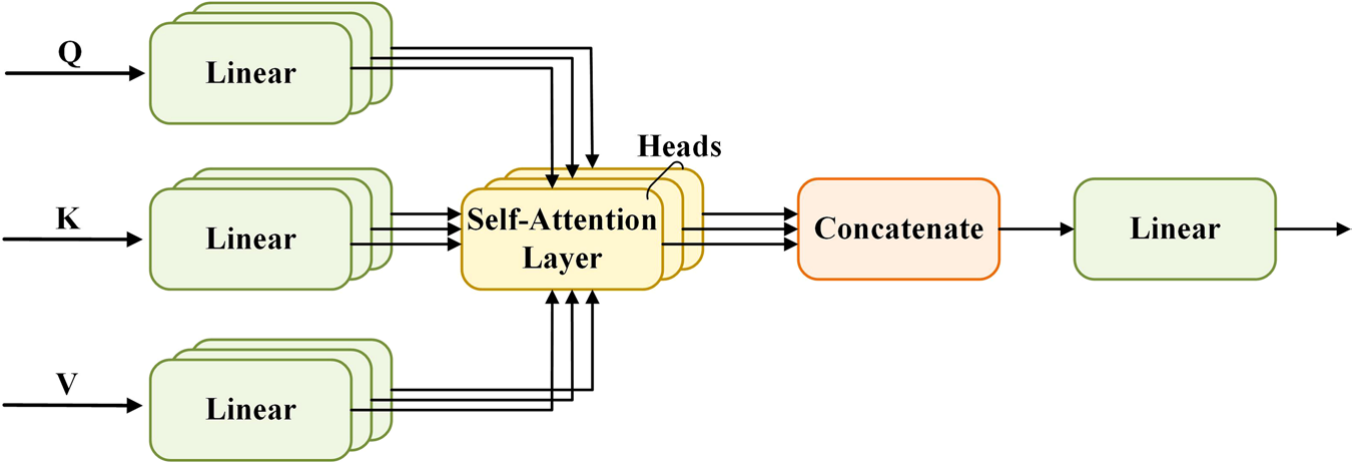
The structure of the multi-head self-attention mechanism.

The transformer model^40^ was a typical encoder-decoder architecture, which is connected by multiple self-attention and stacked by multi-head attention layers, and a feed-forward network connection layer. The multi-head self-attention mechanism is shown in Fig. 3. The attention mechanism efficiently pays attention to the more critical information in the task goal. Multi-head self-attention first needs to set three trainable weight matrices Q, K, and V, and then use the scaled dot-product attention to calculate the self-attention weight

1 $$self \mbox{-} attention(Q,K,V)=Softmax\left(\frac{Q{K}^{T}}{\sqrt{{d}_{k}}}\right)V$$

where, *Q* = *XW*^*Q*^, *K* = *XW*^*K*^, *V* = *XW*^*V*^, *X* is the input feature map with the learnable weight matrix *W*^*Q*^, *W*^*K*^, and *W*^*V*^. *d*_*k*_ is the vector dimension of each key. The attention weight is then connected with multiple self-attention mechanisms to form a multi-head attention mechanism, and its calculation formula is as follows:

2 $$MultiHead(Q,K,V)=Concat(hea{d}_{1},...,hea{d}_{h}){W}^{O}$$

3 $$hea{d}_{i}=self{\rm{ \mbox{-} }}attention({Q}_{i},{K}_{i},{V}_{i})$$

### Swin transformer model

**Fig. 4:**
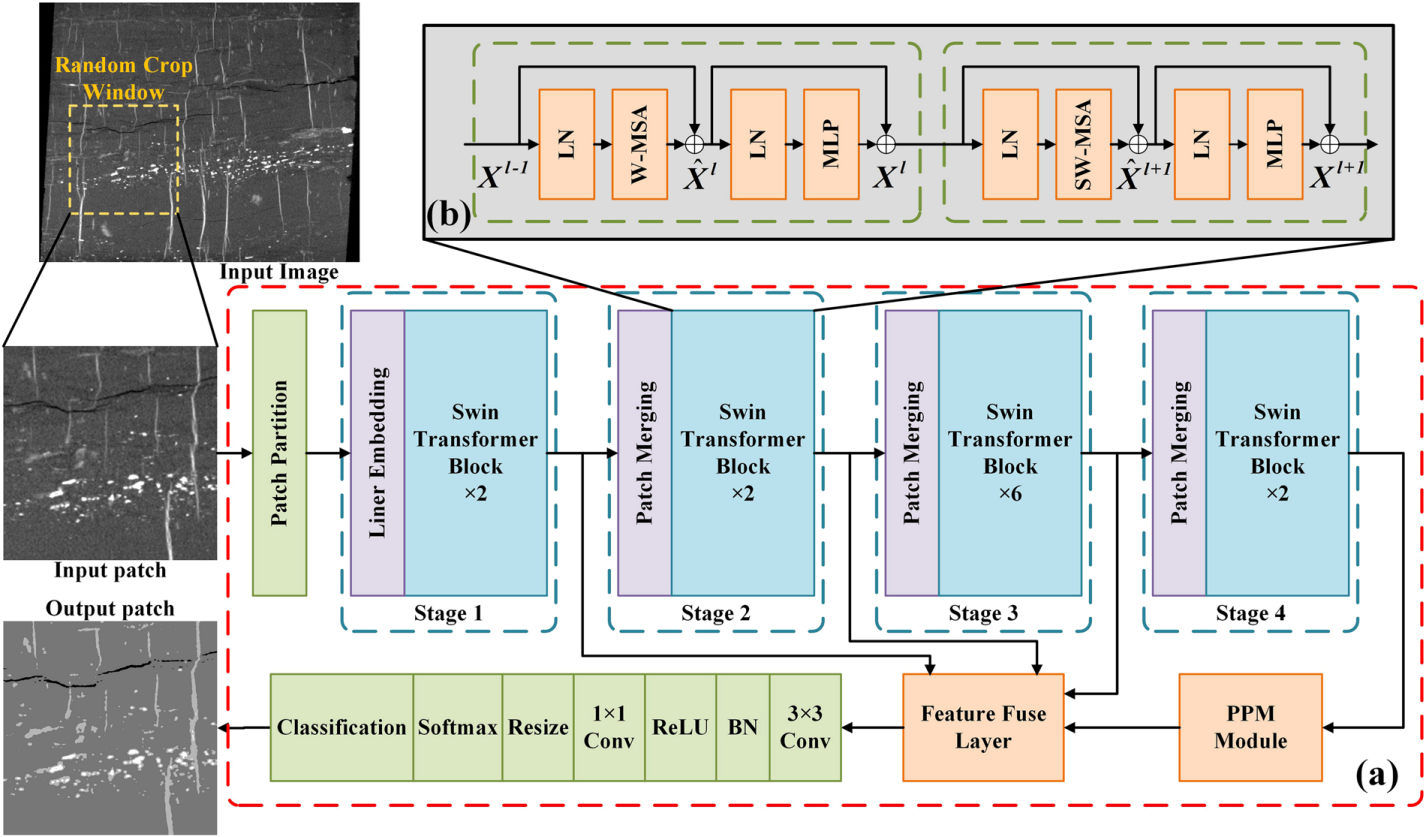
The workflow of the semantic segmentation with the Swin-T network. (**a**) The structure of the Swin-T network (**b**) The structure of two successive Swin-T blocks.

As an improved Transformer model^47,48^, Swin-T has the ability to establish long-range dependencies by using moving windows, which overcomes the shortage of information interaction between groups. As shown in Fig. 4a, the Swin-T model is composed of Patch Partition, Linear Embedding, Swin Transformer Block, and Patch Merging. Each stage reduces the resolution of the input feature map to expand the receptive field layer by layer. The image is first input into the Patch Partition module to divide the image into non-overlapping image blocks, each divided image block is regarded as a token, and the flattening operation is performed in the channel direction. The Linear Embedding module then uses linear variation to map it into a vector of dimension.

**Fig. 5:**
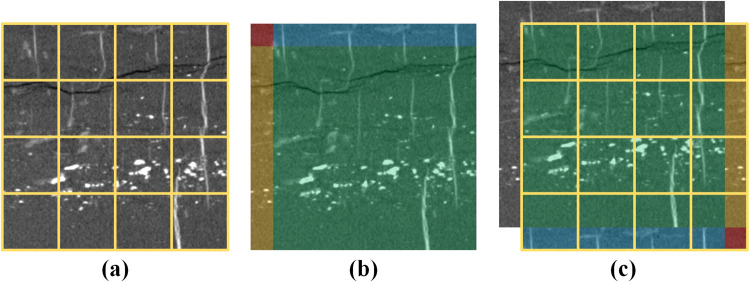
(**a**) Window segmentation of input patch in the W-MSA module; (**b**) Operation of moving window; (**c**) Shifted window segmentation of input patch in the SW-MSA module.

The Swin-T block is a cascade of two multi-head attention modules, consisting of Windowed Multi-Head Self-Attention (W-MSA), Shifted Windowed Multi-Head Self Attention (SW-MSA), and Multilayer Perceptron (MLP). The Layer Norm (LN) layer is used before each MSA module and each MLP that aims to make the training more stable and connected by residual after each module. The W-MSA in the Swin-T model first divides the input image into several non-overlapping windows, and then the pixels in each window is performed with other pixels in the window to obtain information. The SW-MSA mechanism can complete the pixel self-attention calculation of the offset window, thereby indirectly increasing the receptive field of the network and improving the efficiency of information utilization. The MLP module employs a fully connected approach to compute the relationships between each pixel within a fixed-size window. Simultaneously, it utilizes the GELU activation function^59^, thereby enhancing non-linearity performance and network generalization^60–62^. The specific operation is shown in Fig. 5. The features from other Swin-T blocks passed through several convolutional layers, and then fused with the features obtained from PPM. The fused features are used to produce the final segmentation result based on the probability distribution of each pixel in the pores, coal, gangue, and pyrites.

### Training process using Swin-T network model

**Fig. 6:**
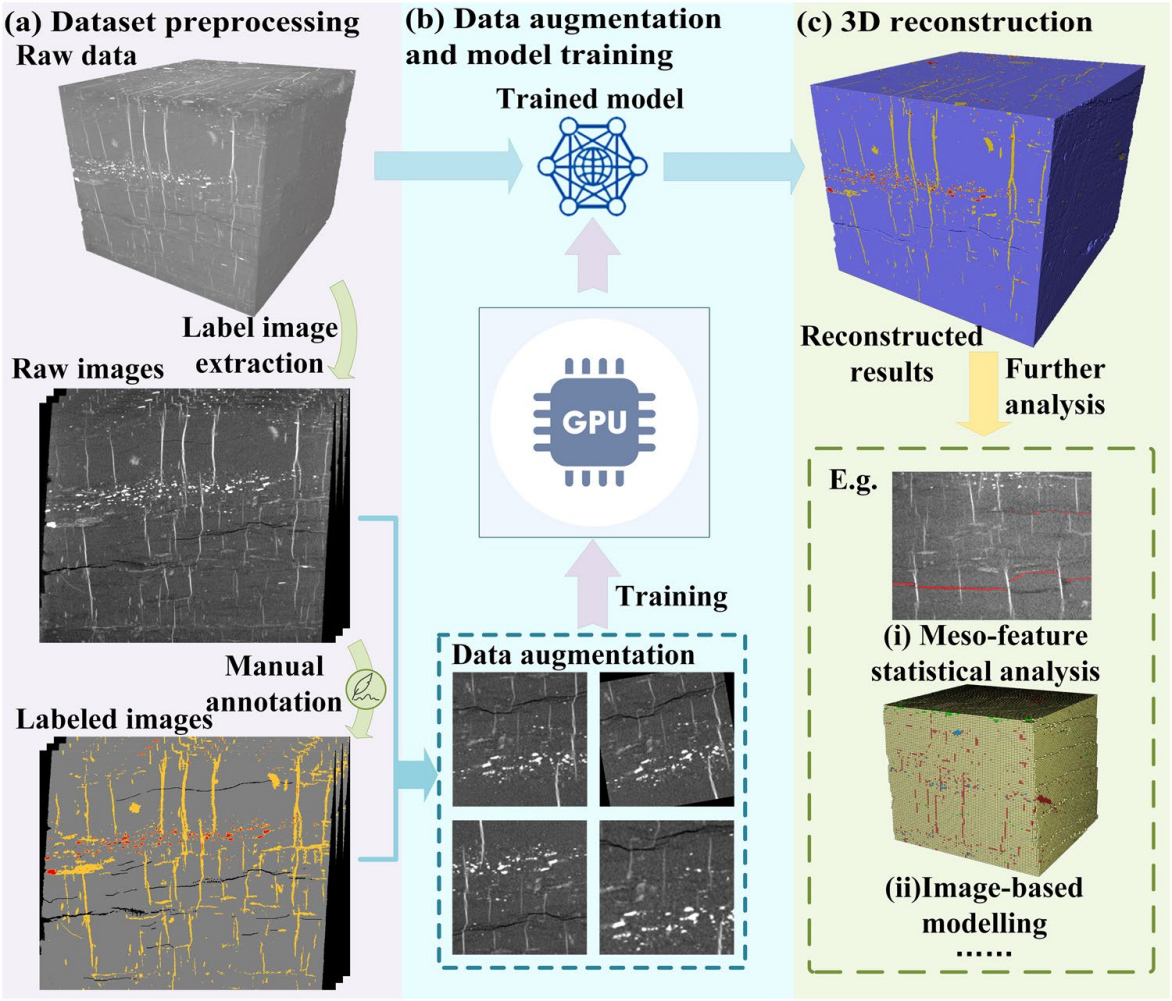
Schematic illustration of (**a**) Dataset preprocessing, (**b**) Data augmentation and model training, (**c**) 3D reconstruction.

The training and testing processes of the Swin-T network is summarized in Fig. 6. Before training, as the size of raw images is 1720 × 1771 pixels with a pixel size of 5.35 μm, the masks are randomly cropped from the full-size images to 448 × 448 sub-images to meet the need of GPU memory. Then, the corresponding masks are fed into the Swin-T to conduct model training processing. This is followed by morphological operations, such as the boundaries and regions enhancement to get the final segmentation. Finally, extensive experiments were carried out to evaluate the performance of the proposed approach, and various loss functions were also developed in combination with training results comparison to supervise the training of the early layers of the multi-task and multi-output network.

The model training was performed on a workstation with a 24GB graphics memory Nvidia RTX 3090TI GPU and CUDA (v11.7) acceleration. The datasets were trained using Swin-T architecture in batches of a user-defined number of images with PyTorch 2.0, and the UNet and DeepLabv3 + architectures were used as comparison groups for the verification of the effectiveness and superiority of the improved mode.

## Data Records

The datasets were made available from figshare https://figshare.com/projects/Raw_data_and_segmented_label_data_of_rock_sample_derived_from_X-ray_CT/162046. There are three items in the project: “Raw XCT data of rock”^63^, which contains a.raw files for rock XCT data; “Training dataset and model weight”^64^, which contains the dataset used for Swin-T training and the well-trained network weight; and “3D representative model”^65^, which contains a .nii file for the 3D reconstructed results obtained from the well-trained Swin-T model.

## Technical Validation

In this section, two important metrics are selected to measure the performance of the entire network: one is accuracy^54^, which is used to measure the percentage of the correctly predicted pixels. The other indicator is the intersection over union (IoU)^55^, which was used to measure the ratio between the intersection and union of predicted and labelled pixel areas.

4 $$IoU=\frac{TP}{TP+FN+FP}$$

5 $$Accuracy{\rm{=}}\frac{TP+TN}{TP+TN+FN+FP}$$

Where, TP, TN, FP, and FN are the true positive, true negative, false positive, and false negative pixel amounts, respectively.

### Evaluation of segmentation models

**Fig. 7:**
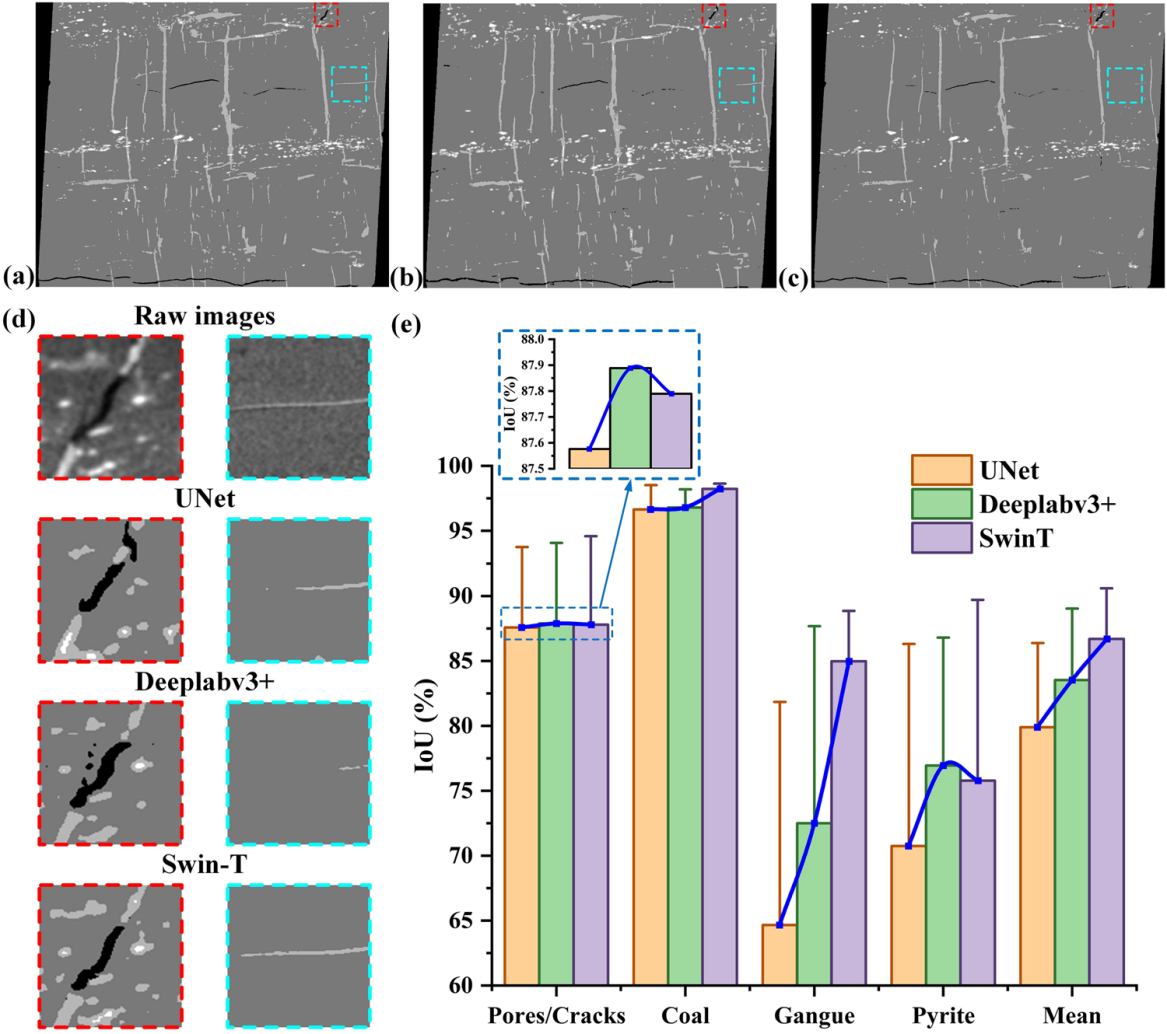
Segmentation results comparison with different networks. (**a**) presents the segmentation result of the Swin-T architecture; (**b**) presents the segmentation results of the U-Net architecture; (**c**) presents the segmentation result of Deeplabv3 + with the ResNet101 backbone (**d**) presents the enlarged view comparison of different networks; (**e**) presents the variation of IoU metric for the whole sample and its internal phases influenced by various networks.

To verify the effectiveness and superiority of the improved model proposed in this paper, the Swin-T model is compared with UNet and DeepLabv3 + architectures with the same dataset, experimental environment and the same network parameter configuration. The effect of various networks on the final IoU index is summarized in Fig. 7. It is clear that the Swin-T model achieves superior segmentation results, with a mean IoU value of 87.63%, significantly higher than the global accuracy values of 80.14% and 84.39% obtained from UNet and DeepLabv3 + respectively. Similar change trend also can be found in coal and gangue phases. However, it should be also noticed that the IoU value obtained from the DeepLabv3 + are slightly higher than the results obtained from the UNet and Swin-T model in crack and pyrite phases, this is mainly due to the crack and pyrite phases were not specifically considered in the ground truth datasets.

For better visual comparison of the selected sample segmentation capabilities of each comparison method, the segmentation results are also visualized in Fig. 7, where the predicting segmentation results of each comparison method are marked by enlarging the interest area of the image. The UNet algorithm often provides unsatisfactory results because of some pixels in the pyrite label is wrongly segmented into gangue phase and some gangue phases are also mis-segmented into cracks. For DeepLabv3 + architecture, the results are slightly improved due to only few coal phases are under-segmentation, which can be further validated by the error bar shown in Fig. 7e. However, Swin-T algorithm works well in the segmentation of the selected samples (Fig. 7d), as Swin-T can automatically find discriminative and representative features by training the model.

### Evaluation of various loss functions

In deep learning approach, the loss function is used to estimate the deviation between the predicted value and the real value of the network. By minimizing the loss function, the model can reach the convergence state and reduce the error of the predicted value of the model. In this section, we proposed a hybrid loss function that contains cross entropy (CE) loss and focal loss (FL)^66^. The hybrid loss function is defined as:

6 $${L}_{CE}(\widehat{p})=-\log (\widehat{p})$$

7 $${L}_{FL}(\widehat{p})=-{\left(1-\widehat{p}\right)}^{\gamma }\log (\widehat{p})$$

8 $${L}_{Hybird}=w\times {L}_{CE}+(1-w)\times {L}_{FL}$$

Where, $\widehat{p}$ ∈ [0, 1] is the model’s estimated probability matrix for the class, γ is tunable focusing parameter, γ ≥ 0, and *w* ∈ [0, 1] is the dynamic coefficient^67^ for the hybrid loss function. the decreasing value based on the training iteration process. Here, *w* gradually decreases from 1 to 0 as iterations increase, resulting in a higher proportion of FL in the loss function and ultimately leading to better network results. The γ is set to 2 that aims to enhance the discrimination among various objects.

**Fig. 8:**
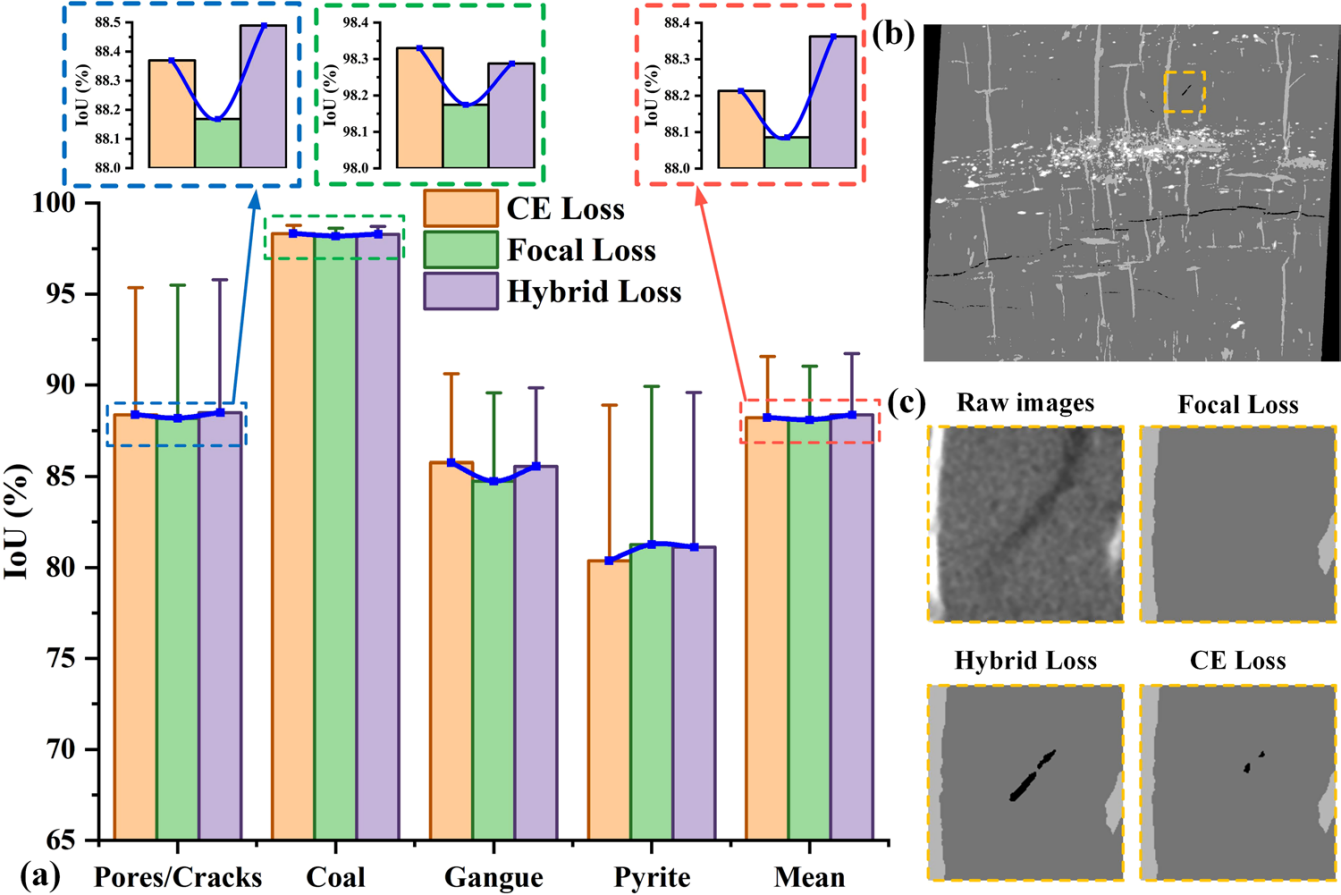
Qualitative and quantitative analysis of the trained network influenced by different loss functions. (**a**) presents the comparison results of the IoU metrics. (**b**) presents the segmentation results with hybrid loss. (**c**) presents the quantitative comparison results influenced by different loss functions.

The effects of various loss functions on the IoU index are shown in Fig. 8. As shown in Fig. 8a, the mean IoU values of the whole sample and its internal phases (including pore, coal, gangue and pyrite) obtained from the hybrid loss function is higher than the results obtained from CE loss function and Focal loss function. We can see that a better segmentation result can be achieved by using the hybrid loss function, with 88.36%, 88.48%, 98.28%, 85.55% and 81.11% for the whole sample, pore, coal, gangue, and pyrite respectively. The proposed hybrid loss function applies a modulating term to the cross-entropy loss, which can effectively discount the effect of easy negatives.

Qualitative analysis shown in Fig. 8b,c also proved that the segmentation effects are significantly improved by using hybrid loss function. As shown in the enlarged images, the cracks existed in the raw images cannot be identified through CE loss and Focal loss, but the pixels in the cracks can be identified more correctly by using the hybrid loss function. The quantitative analysis further proved that the proposed approach is simple and highly effective.

### Evaluation of various patch sizes

**Fig. 9:**
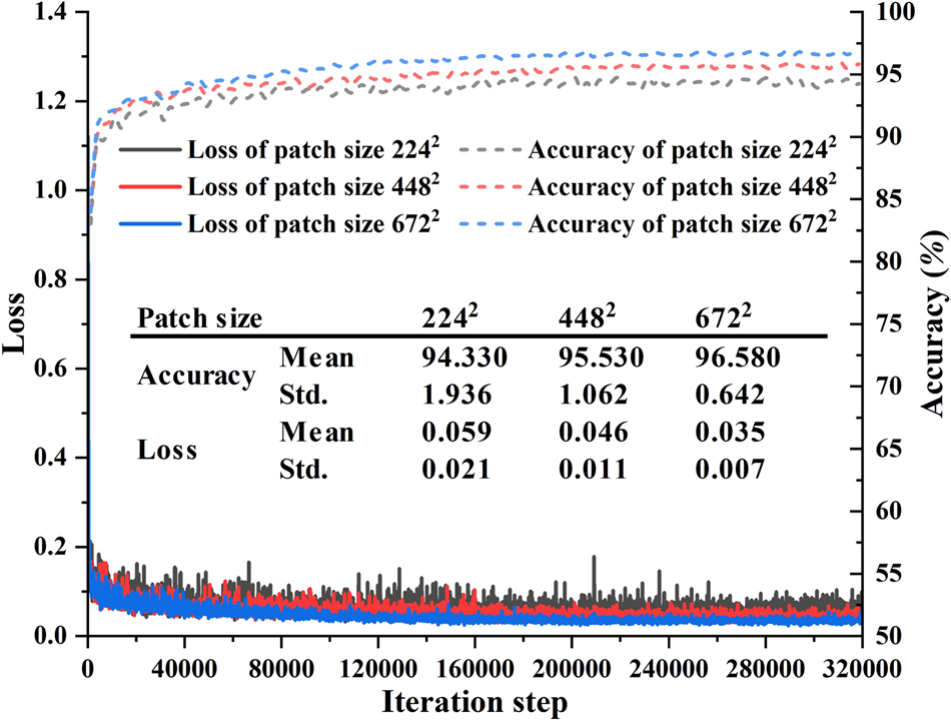
Relationship between the convergence of accuracy, loss and iteration step during the training process.

**Fig. 10:**
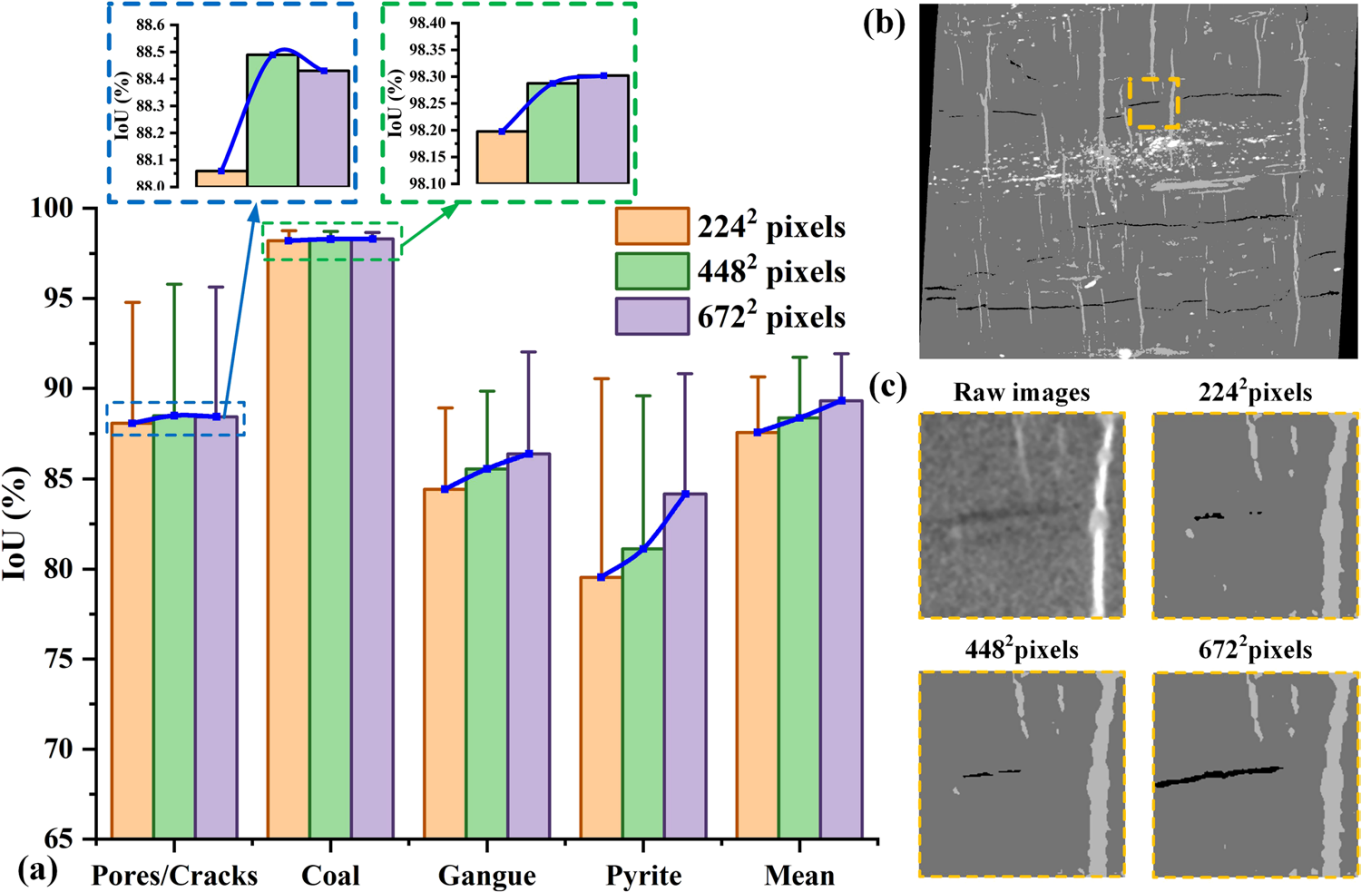
Qualitative and quantitative analysis of the trained network influenced by different patch sizes. (**a**) presents the comparison results of the IoU metrics. (**b**) presents the segmentation results with a patch size of 672^2^ pixels. (**c**) presents the quantitative comparison results influenced by different patch sizes.

In order to compare the impact of iteration times on the training effect influenced by various patch sizes, three patch size (224^2^, 448^2^, and 672^2^) are selected to illustrate model enhancement degree when the model is fully trained and the results are summarized in Figs. 9, 10. It can be clearly seen from the Fig. 9 that the loss values of all networks are continuously decreasing in the initial stage of training, and eventually become stable after 80000 iterations. Especially, the model training convergence is notably superior with a patch size of 672^2^ compared to the other two patch sizes. As shown in Fig. 9, it also can be seen that the average accuracy for a patch size of 672^2^ is 96.58%, with a standard deviation of 0.642. The mean loss value is 0.035, with a standard deviation of 0.007. These findings suggest that using a larger patch size improves the stability of the network’s predictive performance. This leads to a lower probability of prediction errors, allowing the model to achieve higher accuracy values.

Qualitative analysis shown in Fig. 10 proved that a better segmentation result can be achieved using a patch size of 672^2^, with 88.42%, 98.30%, 86.36%, and 89.31% for the pore phase, coal phase, gangue phase and the mean value of the sample respectively, except the pyrite with 84.16%. This is mainly due to the pyrite boundaries are not sufficiently manual labelled in the ground truth data, and the network usually mistake the pyrite and the gangue in composite rock materials. It can be inferred that a larger receptive field allows the network to extract more informative features, resulting in more accurate results. However, it should be noticed that increasing the patch size also brings the drawback of higher computational costs for training process.

### Quantitative characterization of the meso-structure of the selected coal sample

**Fig. 11:**
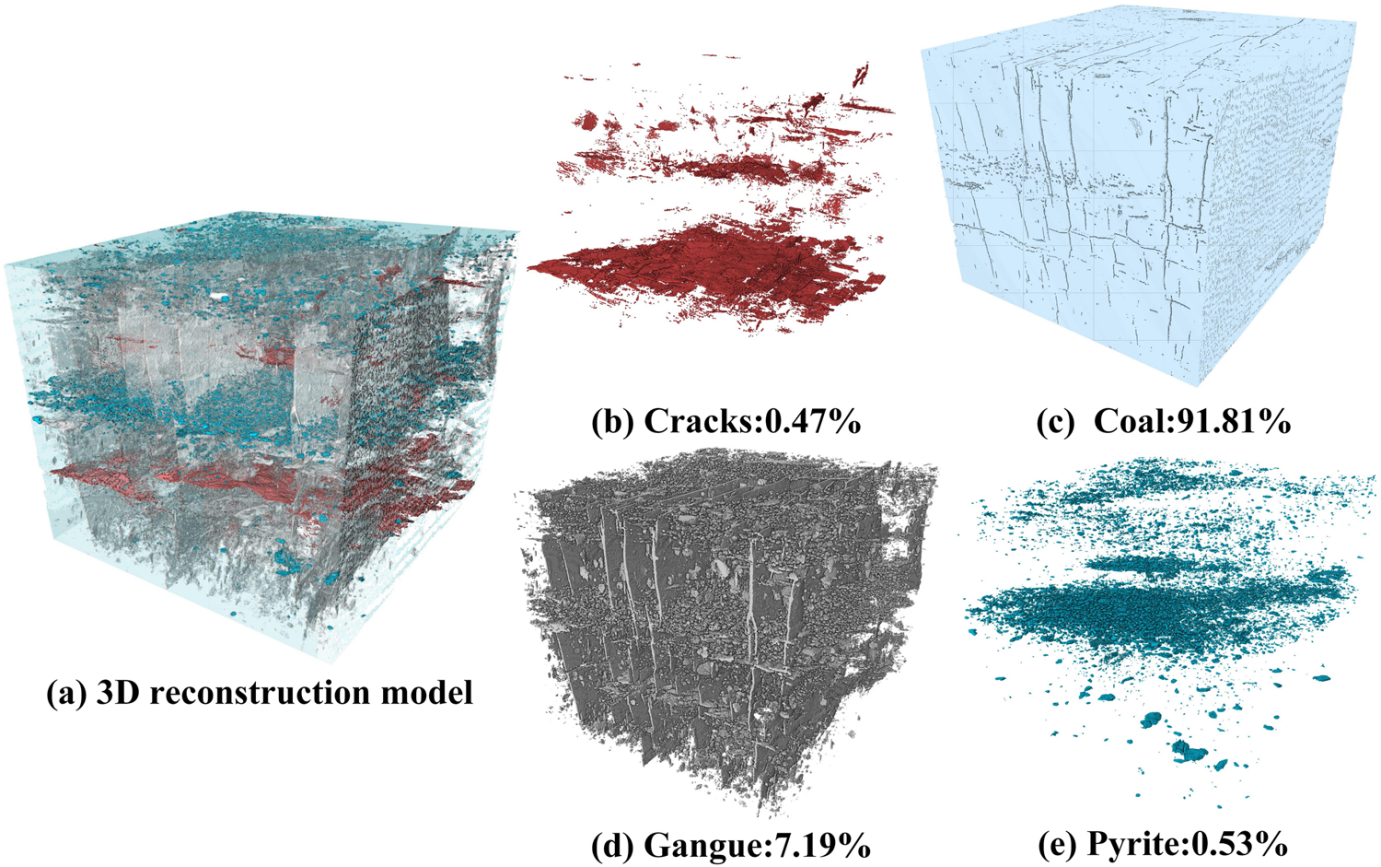
(**a**) 3D volume rendering images of the composite coal rock based on the Swin-T segmentation results (the cracks are in blue and the coal in purple, the gangue in yellow and the pyrite in red); (**b**–**e**) presents the reconstruction model of sub-phases.

To quantitatively characterize internal mineral phases of the selected coal sample, a series of morphological operations are carried to identify the boundary of different mineral phases using the commercial software Avizo 9.0. As shown in Fig. 11, most of the pixels belonging to the coal, gangue, pyrite and cracks were correctly identified, except some micro-cracks and spots. The volume fraction of 0.47% cracks, 91.81% coal, 7.19% gangue, and 0.53% pyrite are quantitatively determined (Fig. 11a–e).

It should be noticed that the real volume fraction for the internal mineral phases is not obtained, the accuracy of various segmentation methods cannot be evaluated quantitatively. However, the comparison of the segmented volumes, as demonstrated through 3D volume rendering, suggests that the segmentation accomplished using Swin-T was satisfactory and exhibited significant enhancement.

## Data Availability

The codes, which are used to generate the 3D representative models and the related results of this manuscript, are released and publicly available at https://github.com/Chall513032/CoalSegmentation.
